# The Effect of Loneliness on Plasma AD Biomarkers in Cognitively Normal Elderly

**DOI:** 10.1002/alz.092634

**Published:** 2025-01-09

**Authors:** Chelsea Reichert Plaska, Davide Bruno, Jaime Ramos Cejudo, Ricardo S. Osorio, Henrik Zetterberg, Kaj Blennow, Nunzio Pomara

**Affiliations:** ^1^ NYU Grossman School of Medicine, New York, NY USA; ^2^ Nathan S. Kline Institute, Orangeburg, NY USA; ^3^ Liverpool John Moores University, Liverpool UK; ^4^ VA Boston Cooperative Studies Program MAVERIC, VA Boston Healthcare System, Boston, MA USA; ^5^ Wisconsin Alzheimer’s Disease Research Center, University of Wisconsin School of Medicine and Public Health, Madison, WI USA; ^6^ Institute of Neuroscience and Physiology, The Sahlgrenska Academy at the University of Gothenburg, Mölndal Sweden; ^7^ UCL Institute of Neurology Queen Square London UK, London UK; ^8^ UK Dementia Research Institute, University College London, London UK; ^9^ Clinical Neurochemistry Laboratory, Sahlgrenska University Hospital, Mölndal Sweden; ^10^ Hong Kong Center for Neurodegenerative Diseases, Clear Water Bay Hong Kong; ^11^ Paris Brain Institute, ICM, Pitié‐Salpêtrière Hospital, Sorbonne University, Paris France; ^12^ Neurodegenerative Disorder Research Center, Institute on Aging and Brain Disorders, University of Science and Technology of China and First Affiliated Hospital of USTC, Heifei China

## Abstract

**Background:**

Social isolation (SI) and loneliness are related to several negative health outcomes, including cognitive decline and dementia. While both males and females experience increased SI as a function of age, studies have found that females experience greater loneliness than males, despite males reporting more physical isolation and smaller social networks. Females, independent of SI‐status, are also at increased risk of Alzheimer’s disease (AD). These sex‐related differences in SI and AD‐risk prompted us to examine whether sex and loneliness influenced the plasma AD biomarkers in a cohort of elderly individuals.

**Method:**

Participants were enrolled in the Memory Education and Research Initiative (MERI) program. MERI participants completed a neuropsychological battery, clinical and psychiatric evaluation, and blood draw. Plasma Aβ40, Aβ42, and PTau231 concentrations were determined using single‐molecule array (SIMOA) platform. Plasma Aβ42/Aβ40 ratio was calculated. MERI participants were included if they gave blood, were cognitively‐normal defined by MMSE>27, age 50 years older, and completed the Profile of Mood States scale (POMS). POMS‐Loneliness item was coded to a dichotomous variable (0‐Not Lonely; 1‐Lonely).

**Result:**

A total of 459 MERI participants (mean age=71 years; 59% females) were included. There were no significant differences between males and females for education, HAM‐D total score, or MMSE, only age. A 2x2 analysis of covariance (ANCOVA) with sex (male, female) and loneliness (not lonely, lonely), and age as a covariate, revealed a significant interaction between sex and loneliness with Aβ42 (p=0.014). Lonely females had significantly lower Aβ42 than males who are lonely. The same analysis revealed a significant main effect of sex on Aβ40 (p=0.014) and PTau231 (p=0.007); females had higher Aβ40 and lower PTau231, respectively. There were no significant main effects or interactions for Plasma ratio Aβ42/Aβ40.

**Conclusion:**

Cognitively‐normal females who reported being lonely, as compared with males, had lower levels of plasma Aβ42 but not lower levels of plasma Aβ42/Aβ40 ratio, a better predictor of a positive amyloid PET scan. Future studies should examine if the results reflect increased brain amyloid deposition in lonely females. If so, interventions that mitigate loneliness may reduce overall risk for AD in females.

